# Transcriptome Profiling Reveals a Divergent Adaptive Response to Hyper- and Hypo-Salinity in the Yellow Drum, *Nibea albiflora*

**DOI:** 10.3390/ani11082201

**Published:** 2021-07-25

**Authors:** Xiang Zhao, Zhicheng Sun, Tianxiang Gao, Na Song

**Affiliations:** 1 The Key Laboratory of Mariculture, Ocean University of China, Ministry of Education, Qingdao 266003, China; zx15965582296@163.com (X.Z.); zhichengsun@yeah.net (Z.S.); 2 Fishery College, Zhejiang Ocean University, Zhoushan 316022, China

**Keywords:** functional genomics, salinity stress, global warming, differentially expressed genes (DEGs), illumina sequencing

## Abstract

**Simple Summary:**

Global warming and certain climate disasters (typhoon, tsunami, etc.) can lead to fluctuation in seawater salinity that causes salinity stress in fish. The aim of this study was to investigate the functional genes and relevant pathways in response to salinity stress in the yellow drum. Genes and pathways related to signal transduction, osmoregulation, and metabolism may be involved in the adaptive regulation to salinity in the yellow drum. Additionally, the genes under salinity stress were mainly divided into three expression trends. Our results provided novel insights into further study of the salinity adaptability of euryhaline fishes.

**Abstract:**

The yellow drum (*Nibea albiflora*) is an important marine economic fish that is widely distributed in the coastal waters of the Northwest Pacific. In order to understand the molecular regulatory mechanism of the yellow drum under salinity stress, in the present study, transcriptome analysis was performed under gradients with six salinities (10, 15, 20, 25, 30, and 35 psu). Compared to 25 psu, 907, 1109, 1309, 18, and 243 differentially expressed genes (DEGs) were obtained under 10, 15, 20, 30, and 35 psu salinities, respectively. The differential gene expression was further validated by quantitative real-time PCR (qPCR). The results of the tendency analysis showed that all DEGs of the yellow drum under salinity fluctuation were mainly divided into three expression trends. Gene Ontology (GO) and Kyoto Encyclopedia of Genes and Genomes (KEGG) pathway enrichment analysis showed that the PI3K-Akt signaling pathway, Jak-STAT signaling pathway as well as the glutathione metabolism and steroid biosynthesis pathways may be the key pathways for the salinity adaptive regulation mechanism of the yellow drum. G protein-coupled receptors (GPCRs), the solute carrier family (SLC), the transient receptor potential cation channel subfamily V member 6 (TRPV6), isocitrate dehydrogenase (IDH1), and fructose-bisphosphate aldolase C-B (ALDOCB) may be the key genes in the response of the yellow drum to salinity stress. This study explored the transcriptional patterns of the yellow drum under salinity stress and provided fundamental information for the study of salinity adaptability in this species.

## 1. Introduction

Salinity is an inherent physicochemical property of seawater, and fluctuations in salinity can affect the growth, reproduction, feeding, endocrine, and metabolism of marine organisms [1,2,3,4,5]. Most marine fish are always immersed in seawater, and their behavior and internal physiological states are particularly sensitive to environmental salinity changes [6]. Fluctuations in the salinity of a water environment cause salinity stress in fish and can interfere with the homeostasis and normal physiological process of their internal environment. For example, an increase of salinity may have a negative impact on white blood cell count (WBC) of fish [7,8]. Global warming has been gradually raising the sea level and therefore reducing salinity [9]. In addition, certain types of climate disasters (typhoon, tsunami, etc.) have led to fluctuations in seawater salinity in coastal areas. These salinity fluctuation events cause salinity stress in marine organisms which may eventually exceed the tolerance limit of some species [10]. Euryhaline fish can adapt to large salinity changes compared to stenohaline fish [11], and therefore, they can be used as an excellent model for studying the adaptive regulation mechanism of marine organisms to salinity stress.

The yellow drum (*Nibea albiflora*) belongs to the Sciaenidae, Perciformes. It is a euryhaline fish with obvious seasonal migration habits and is naturally distributed in the coastal waters of China, Japan, and Korea [12]. Fluctuations in the salinity of coastal areas caused by natural disasters and river runoff may contribute to the yellow drum’s high tolerance to salinity stress. Previous studies have shown that the yellow drum can survive in a salinity range from approximately 14 to 34 psu [13], and results of preliminary experiments in this study showed that the yellow drum can even survive in a salinity range from 10 to 35 psu. Therefore, the yellow drum could be an excellent model for studying the response mechanism of marine fish to salinity stress. In addition, the yellow drum is an important mariculture fish. The extreme environmental salinity caused by global climate change poses a huge challenge to yellow drum farming [14]. Elucidating the potential mechanism of the yellow drum adapting to environmental salinity fluctuation could aid aquaculture and improve our understanding of the osmotic regulation ability of euryhaline fishes.

In recent years, transcriptome sequencing has played an important role in exploring the complete structure of a particular cell or organism at a particular stage of development or under physiological conditions [15,16]. It is a stable and reliable method for identifying important physiological pathways in which organisms respond to various conditions. It is also helpful to explore the changes of gene expression caused by abiotic stress [17,18]. For example, different doses of natural feed components may affect the expression of some genes in fish. Transcriptome analysis shows that low doses of natural feed components can increase the expression of immune and growth-related genes in fish [19,20,21]. In addition, the transcriptome analysis of cobia (*Rachycentron canadum*) under salinity stress showed that metabolism was the pathway that was the most affected by salinity changes [22]. Sun et al. found that the DEGs related to the adaptive regulation mechanism of salinity in *Acanthogobius ommaturus* were mainly involved in the metabolic, ion transport, and signal transduction processes [23]. Several genes of the solute carrier (SLC) gene family have been identified as candidate genes that mediate the response of the tiger puffer (*Takifugu rubripes*) to low-salinity stress [24]. Tian et al. investigated the salinity adaptability of the yellow drum based on growth performance, blood ion concentrations, enzyme activities, and intestinal microbiota composition. The results showed that the yellow drum showed good adaptation to moderately low salinities [25]. However, adaptive regulatory pathways and the genes of the yellow drum in response to salinity stress are still largely unexplored.

In the present study, we determined the transcriptome changes that occur in response to different salinity gradients in the gill of the yellow drum. The results of this study could be a valuable resource for understanding the mechanisms of salinity adaptation and for promoting genetic studies in the yellow drum.

## 2. Materials and Methods

### 2.1. Ethics Statement

The yellow drum is not an endangered species. We anesthetized it with eugenol in field experiments to minimize the pain of all of the sample fish. All animal experiments were conducted in accordance with the Guidelines for Experimental Animals of the Ministry of Science and Technology (Beijing, China, no. 398, 30 September 2006).

### 2.2. Experimental Fish and Salinity Stress Program

The experimental fish (10.1 ± 1.8 g) were from the Aoshanwei National Marine Research Center, Qingdao City, Shandong Province, China. The fish samples used in this study had been cultured in the farm using the natural seawater (temperature: 20 °C, salinity: 31 psu and pH: 7.5) since they were bred. In the manner of acute stress, the animals were placed in six separate aquariums (80 cm× 60 cm× 40 cm) with 9 fish per aquarium, each with a salinity gradient. The six salinities were 10, 15, 20, 25, 30, and 35 psu. Among them, 25 psu was used as the control group, and the other salinities were used as the treatment groups. A total of five groups of experiments with salinities of 10 vs. 25, 15 vs. 25, 20 vs. 25, 30 vs. 25, and 35 vs. 25 were analyzed. After 24 h of salinity stress, all samples were active, and no samples died. A total of three experimental individuals of similar size, vigor, and health were immediately euthanized and sampled in each experimental group. Gill tissues were collected and quickly frozen in liquid nitrogen for RNA extraction.

### 2.3. RNA Extraction and Illumina Sequencing

In this study, total RNA of the samples was extracted using a standard Trizol Reagent Kit following the manufacturer’s protocols. The extracted total RNA was then diluted according to a certain proportion before the concentration and integrity test. The RNA concentration was measured using a NanoDrop 2000 (Thermo Scientific, Waltham, MA, USA). RNA integrity was assessed using an Agilent RNA 6000 Nano Kit from the Agilent Bioanalyzer 2100 system (Agilent Technologies, Santa Clara, CA, USA), and only high-quality samples (OD260/280 ≥ 1.8 and OD260/230 ≥ 1.8) were used to build a library for sequencing.

The mRNA with poly-A tail enriched with magnetic beads with Oligo dT was used to fragment the RNA using interrupting buffer. Random N6 primers were reverse transcribed, and then double-stranded cDNA was synthesized to form double-stranded DNA. The ends of the synthesized double-stranded DNA were flattened and phosphorylated at the 5’end, forming a sticky end protruding “A” at the 3’end, and then connecting a bubble-like joint with a protruding “T” at the 3’end. The ligated products were amplified using PCR with specific primers. The PCR product was thermally denatured into a single strand, and then the single strand DNA was cyclized with a segment of bridge primers to obtain a single strand cyclic DNA library. Sequencing was then carried out using the GooalGene technology Company (Wuhan, China) on the Illumina Hiseq Nova platform. The raw data files were passed to the NCBI’s Sequence Read Archive (SRA) (GenBank accession no. PRJNA736181).

### 2.4. Data Filtering and Mapping

The raw sequencing data contained readings of low quality, combined contamination, and high unknown base N content. These reads needed to be filtered before subsequent analysis to ensure the reliability of the results. In this study, FASTP software was used for filtering [26]. First, the reads containing the joint were removed, the reads with an unknown base N content greater than 10% were then removed, and finally, the low-quality reads were removed. The filtered “clean reads” were saved in FASTQ format. We then used HISAT software [27] to map the clean reads to the reference genome sequence (GenBank accession no. GCA_900327885.1). After mapping with the reference genome, we used rMATS software to detect the alternative splicing of the sample [28]. In addition, GATK software was used to detect the SNP and indel information of each sample [29].

### 2.5. Differential Expression Genes Analysis and Functional Annotation

To investigate the differential responses of the yellow drum to salinity fluctuations, we analyzed the number and biological functions of DEGs in the five treatment groups. First, we mapped all of the clean reads onto the reference gene sequence using Bowtie2 [30]. The gene expression levels of each sample were then calculated to determine the fragments per kilobase of exon model per million mapped fragments (FPKM) using RSEM with default settings [31].

According to the gene expression level of each sample, DEseq2 software was used to detect significant DEGs, the |log_2_FC| ≥ 1 and Q-value (Adjusted *p* value) ≤ 0.05 were used as the filtering thresholds [32,33]. The DEG expression tendency analysis was performed using Short Timeseries Expression Miner software (STEM) on the OmicShare tools platform [34], a free online platform for data analysis (www.omicshare.com/tools, accessed on 24 July 2021). In addition, in order to further understand the function of differential genes under salinity fluctuations, we classified 3586 DEGs using Gene Ontology (GO) terms and Kyoto Encyclopedia of Genes and Genomes (KEGG) pathway analysis. At the same time, we used the phyper function in R software for the enrichment analysis, and the GO terms and KEGG pathway analysis of Q-value ≤ 0.05 were regarded as significant enrichment.

### 2.6. Quantitative Real-Time PCR Validation

In order to verify the reliability of transcriptome data, we randomly selected eight differentially expressed gene fragments for qRT-PCR analysis based on the differential gene analysis. The specific gene primers were designed by Primer Premier 6 software, and the gene and primer information are shown in Table S1. In addition, β-actin (F: 5′-CCTCCCTGGAGAAGAGCTATGAG-3′; R: 5′-CGCACTTCATGATGCTGTTGTAG-3′) and GAPDH (F: 5′-ACTGTCACTCCTCCATCTT-3′; R: 5′-GGTTGCTGTATCCGAACT-3′) were selected as reference genes for internal standardization. The total RNA used in the qRT-PCR experiment was the same as that used in Illumina sequencing. We used HiScript^®^ IIQ RT SuperMix for qPCR kit (Vazyme, China) to reverse transcribe the total RNA of 15 samples to obtain the cDNA template used in the qRT-PCR experiment. According to the results of the standard curve, 18 cDNA samples were diluted 20-fold in nuclease-free water as templates for qRT-PCR. The QRT-PCR experiment was designed according to the instructions of the SYBR^®^ PreMix Ex TaqTM (Tli RNAse H Plus) RR420A and the StepOnePlus (Takara, Japan). A 20 μL reaction system was amplified, including 10 μL of TB Green^®^ Premix Ex TaqTM (2×), 2 μL of cDNA template, 0.4 μL of forward and reverse primers, and ROX Reference Dye (50×) and 6.8 μL of RNase-free water. The amplification processes consisted of a holding stage of 30 s at 95 °C followed by 40 cycles of 5 s at 95 °C and 30 s at 60 °C. Three parallel experiments on each cDNA template were performed to reduce the error of the experimental results. The relative expression of each the eight genes was analyzed with the comparative cycle threshold (2^−ΔΔCT^) method (∆CT = CT_target gene_ − CT_reference gene_, ∆∆CT = ∆CT_treatment_ − ∆CT_control_).

## 3. Results

### 3.1. Data Filtering and Transcriptome Assembly

After filtering the raw data obtained from Illumina sequencing, about 854 million high-quality clean reads were produced. The quality indicators of the filtered reads are shown in Table S2. All high-quality clean reads were mapped to the yellow drum genome. The mapping rate of the clean reads for each sample was greater than 90%, and a total of 21,642 genes were assembled. After mapping with the reference genome, we detected alternative splicing information for each sample, and almost 90% of the alternative splicing events came from skipped exon (SE). We detected that more than 64% of the SNP types belonged to C-T and A-G. In addition, we also detected the indel information of each sample, and the results showed that nearly 50% of the indel were within the downstream 2000 bp of the genes.

### 3.2. Gene Expression Quantification and Analysis of Differentially Expressed Genes (DEGs)

**Figure 1:**
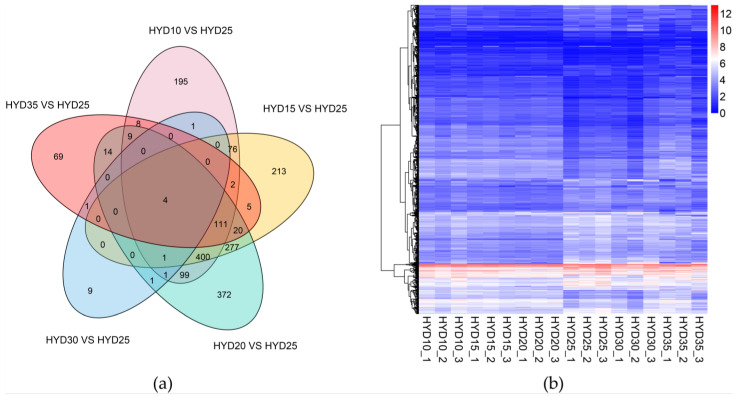
(**a**) The global overview of differentially expressed genes based on a Venn diagram. (**b**) Clustering heat map of differentially expressed genes. The horizontal axis represents the log_2_ (FPKM + 1) of the sample, and the vertical axis represents the gene. The redder the color is, the higher the expression, and the bluer the color is, the lower the expression.

We mapped clean reads to gene sequences and used RSEM software to calculate the gene amount of each sample. In order to explore the gene expression pattern of the yellow drum under salinity fluctuation, we compared the number of DEGs in five treatment groups. Compared to the control (25 psu), 907 (471 up- and 436 down-regulated), 1109 (498 up- and 611 down-regulated), 1309 (627 up- and 682 down-regulated), 18 (7 up- and 11 down-regulated), and 243 (141 up- and 102 down-regulated) DEGs were obtained under 10, 15, 20, 30, and 35 psu salinities, respectively. We constructed a cluster heat map to show the expression of the DEGs in different samples (Figure 1).

### 3.3. Tendency Analysis of DEGs

**Figure 2:**
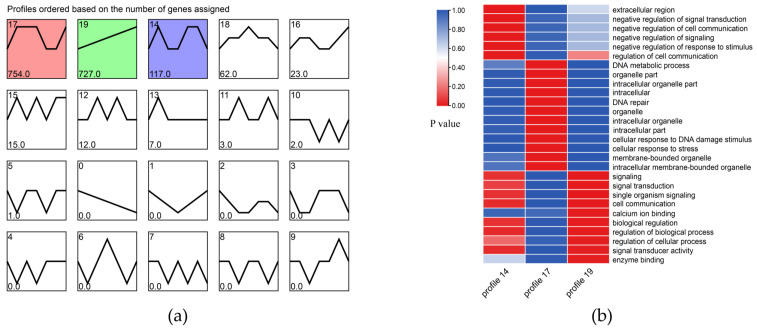
(**a**) Tendency analysis of all DEGs. The trend graph shows three trends of genetic changes. Each number in the upper left corner represents a trend, each number in the lower left corner represents the gene number for each trend type, and colors represent a significant trend (*p* < 0.05). (**b**) GO enrichment graph of genes in three trends.

To explore the expression pattern of the DEGs in the process of salinity fluctuation, we conducted tendency analysis based on the expression quantity data of all DEGs. The results showed that all DEGs under salinity fluctuation were mainly divided into three expression trends (Profiles 14, 17, and 19). In addition, we performed GO enrichment analysis to understand the main functions of the genes in the three trends (Figure 2).

### 3.4. GO Terms and KEGG Pathways Enrichment Analysis of DEGs

**Figure 3:**
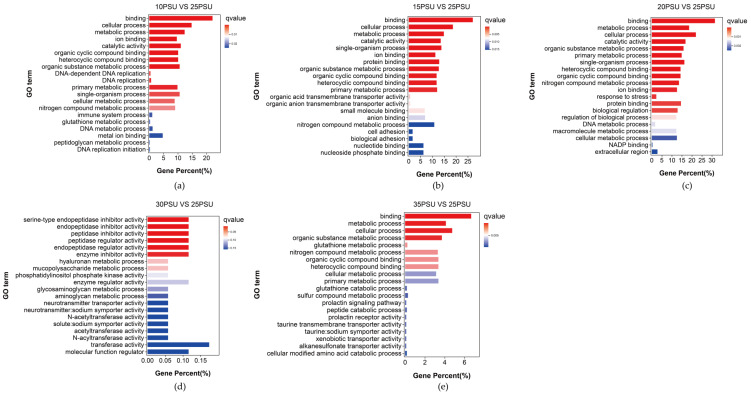
The results of the GO enrichment analysis of the DEGs in the yellow drum under salinity fluctuations. (**a**) 10PSU vs. 25PSU; (**b**) 15PSU vs. 25PSU; (**c**) 20PSU vs. 25PSU; (**d**) 30PSU vs. 25PSU; (**e**) 35PSU vs. 25PSU.

To explore the function of genes that may be related to the response of the yellow drum to salinity fluctuations, we conducted GO and KEGG pathway enrichment analyses for the DEGs of five treatment groups. The results showed that the main GO terms for the DEGs included binding (GO:0005488), cellular processes (GO:0009987), metabolic processes (GO:0008152), and catalytic activity (GO:0003824) (Figure 3).

**Figure 4:**
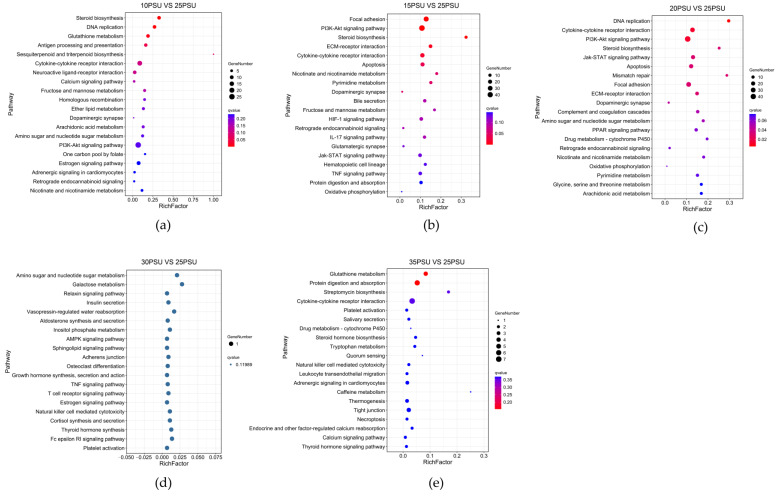
The results of the KEGG pathway enrichment analysis of DEGs in the yellow drum under salinity fluctuations. (The size of the bubble indicates the number of genes enriched to a KEGG pathway, the color represents the Q-value, and the redder the color is, the smaller the Q-value). (**a**) 10PSU vs. 25PSU; (**b**) 15PSU vs. 25PSU; (**c**) 20PSU vs. 25PSU; (**d**) 30PSU vs. 25PSU; (**e**) 35PSU vs. 25PSU.

The KEGG pathway enrichment analysis provided valuable information for the study of specific cellular processes, environmental information processing, genetic information processing, and the metabolism organismal systems of the yellow drum under salinity fluctuations. With an increase in salinity, the DEGs were enriched in 308, 410, 483, 4, and 90 different pathways at 10, 15, 20, 30, and 35 psu, respectively. Notably, the 3, 6, and 11 pathways (Q < 0.05) were significantly enriched at 10, 15, and 20 psu, respectively, while no significant enrichment pathways were found in the 30 and 35 psu. The results are shown in Figure 4.

### 3.5. Validation of Transcriptomic Data via qRT-PCR

**Figure 5:**
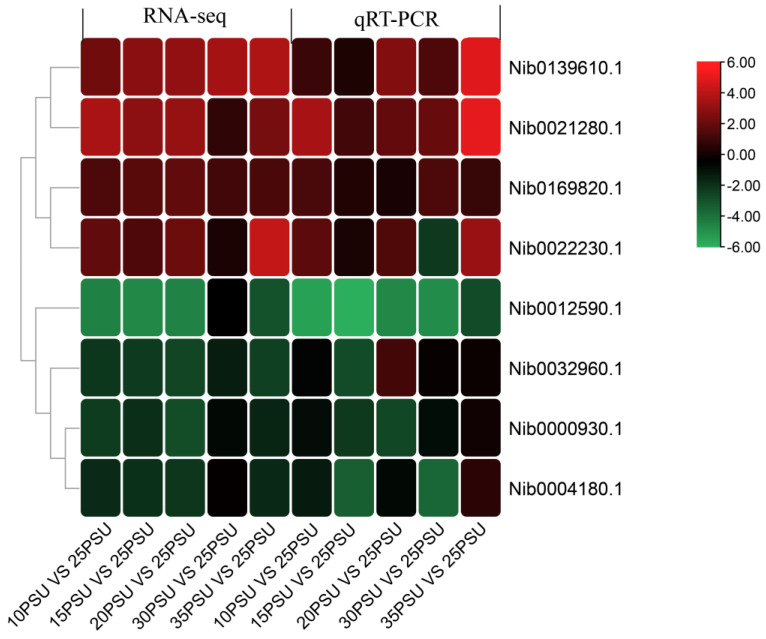
The relative change of the transcriptomes data and qRT-PCR data of eight DEGs.

A total of eight DEGs were randomly selected for the qRT-PCR experiment on 18 cDNA templates. The melting curve analysis showed that a single product was amplified for all of the tested genes, indicating that the transcriptome assembly was largely accurate. The relative expression levels of the eight genes in the five treatment groups compared to the control group are shown in Figure 5. The results showed that all of the tested genes were consistent with the expression patterns of the transcriptome analysis, with only slight differences in the expression levels. In general, the results of the qRT-PCR experiment confirms that the results of the RNA-Seq expression analysis are reliable and accurate.

## 4. Discussion

In the process of long-term evolution, to adapt the adverse effects of salinity changes, euryhaline fish have developed some highly conservative physiological mechanisms so that their internal osmotic pressure can quickly keep balance with the ambient environment [6]. Transcriptome analysis via high-throughput sequencing technologies has been accepted as a robust approach to assess transcriptional response to different salinity conditions [35]. Therefore, transcriptome analysis can provide theoretical guidance for the selection of the optimal salinity in aquaculture.

### 4.1. Expression Patterns of DEGs

To further understand the expression patterns of the DEGs of the yellow drum under salinity fluctuations, we performed a tendency analysis for all of the DEGs and found that (1) the results of the GO enrichment analysis of the genes in profile 14 showed that these genes were mainly related to the negative feedback mechanism of signal transduction. Related studies have shown that the negative feedback regulation mechanism of fish is mainly controlled cell proliferation and immune and stress signal processing [36,37]. Compared to 25 psu, the yellow drum may not activate this negative feedback mechanism under salinity stress in a small range (20 psu and 30 psu); therefore, we observed that the expression trend of these genes was stable or inhibited. Under extreme salinity stress, the body reaction of the yellow drum was intense, and the activation of the negative feedback regulation mechanism may prevent the body from hyperimmunization. (2) The genes in profile 17 were activated under high salinity and low salinity stress. These genes mainly related to DNA metabolism and cell response to stress signals. The salinity stress may cause damage to macromolecules such as DNA and protein, which may be the molecular osmosensors of fish [38]. The damaged macromolecules (DNA and protein, etc.) caused by salinity stress may be the osmotic regulation activators of the yellow drum. (3) The expression trend of genes in profile 19 was positively correlated with the change of salinity. These genes mainly related to signal transduction and osmotic regulation. Genes in this category were very sensitive to salinity fluctuations. Whenever they respond positively to changes in salinity, these intermediate products may be necessary for the basic physiological activities of the yellow drum [39].

### 4.2. Signal Transduction

The response mechanism of fish to salinity stress is the synergistic effect of various stress signal transduction processes. In this study, the PI3K-Akt and Jak-STAT signaling pathways were significantly enriched under low salinity stress. The PI3K-Akt signaling pathway is inferred to be activated by many types of cellular stimuli and regulates fundamental cellular functions such as transcription and translation, etc. [40]. The Jak-STAT signaling pathway is an upstream pathway regulating immune and osmoregulation [41]. The yellow drum may perceive the signal change of osmotic pressure through the PI3K-Akt and Jak-STAT signaling pathways, thus activating the mechanism of osmotic regulation.

G protein-coupled receptors (GPCRs) is a general term for a large class of membrane protein receptors, which are involved in many cellular signal transduction processes. In these processes, GPCRs can bind to certain signal molecules and activate a series of signal pathways in cells, resulting in changes in the state of the cells [42]. In this study, we found that many GPCRs were significantly up-regulated under high salinity and low salinity stress, such as G protein-coupled receptor 87 (GPCR87), cannabinoid receptor 1 (Cnr1), and chemokine XC receptor 1 (Xcr1). GPCR87 is a receptor for lysophosphatidic acid (LPA). A previous study suggested that GPR87 was necessary in response to DNA damage [43]. Salinity stress causes DNA damage, and the damaged DNA may activate the osmotic regulation of the yellow drum through GPCR87. Cannabinoid receptor 1 (Cnr1) is the G protein coupled receptor for cannabinoids. Cnr1 can regulate the osmotic stress response of zebrafish, which suggests that Cnr1 may also play an important role in the osmotic stress signal transduction process of the yellow drum [44]. Xcr1 is a receptor for chemokines SCYC1 and SCYC2. A previous study suggested that Xcr1 transduced signals by increasing the intracellular calcium ion level [45]. Therefore, Xcr1 may be an important signal transduction molecule in the osmotic regulation mechanism of the yellow drums. In summary, GPCRs may play an important role in osmotic stress sensing and signal transduction of yellow drums, and therefore, there is a need for in-depth study.

### 4.3. Osmoregulation

Ion transporters and the permeability of the plasma membrane are of great significance to the osmotic regulation mechanism of euryhaline fish [46,47]. This study focused on several traditional ion transporters and channels. The solute carrier family (SLC) has been shown to be involved in the transport of various ions, proteins, and amino acids in the osmotic regulation of some fish [24,48]. In this study, we observed that the expression of the SLC25A24 and SLC43A2 genes were up-regulated in low salinity stress. SLC25A24 is a calcium-dependent mitochondrial solute carrier and mediates the reversible, electroneutral exchange of Mg-ATP or Mg-ADP against phosphate ions, catalyzing the net uptake or efflux of adenine nucleotides across the mitochondrial inner membrane [49]. SLC43A2 can mediate the movement of bulky neutral amino acids across cell membranes [50]. Therefore, the yellow drum may rely on transporters and accumulate intracellular permeants, including taurine, myo-inositol, amino acids, ions, etc., to balance any osmotic change during changes in salinity. SLC12A3, an electroneutral sodium and chloride ion cotransporter, was up-regulated under low salinity stress and down-regulated under high salinity stress [51]. Therefore, SLC12A3 may be the key mediator of sodium and chloride reabsorption in the yellow drum. In addition, transient receptor potential cation channel subfamily V member 6 (TRPV6), a calcium selective cation channel, was up-regulated under low salinity and high salinity stress [52]. TRPV6 may mediate Ca^2+^ uptake in the yellow drum when under salinity stress.

### 4.4. Metabolism

The stress caused by salinity changes was related to an increase in reactive oxygen species (ROS), which may lead to oxidative damage [53]. The glutathione metabolic pathway is a key pathway in the cell detoxification system, which protects cells from ROS damage [54]. Hence, it was not surprising that the glutathione metabolic pathway was significantly enriched under low salinity stress in this study. Salinity stress responses are energy-demanding processes in fish and require a redistribution of energy [55]. One of the key features of fishes under salinity stress is the redirection of metabolic energy from normal cellular processes to functions related to osmotic regulation mechanisms [56]. In this study, we observed that steroid biosynthesis pathway was significantly enriched under low salinity stress. The yellow drum may use lipids as an energy source under low salinity stress. In addition, we observed the that several carbohydrate metabolism genes such as isocitrate dehydrogenase (IDH1) and fructose-bisphosphate aldolase C-B (ALDOCB) were significantly up-regulated under low salinity stress. These metabolic pathways and genes may provide a lot of available energy for the yellow drum to cope with low salinity stress [57,58]. However, we did not observe any significant enrichment of the pathways related to energy metabolism at 30 and 35 psu, which may imply that 30 and 35 psu did not result in significant stress to the yellow drum.

## 5. Conclusions

RNA-seq is a stable and reliable method for identifying important physiological reactions in which fishes respond to salinity stress. In this study, we performed an RNA-Seq analysis of yellow drum exposed to six salinity gradients, and abundant DEGs were detected. The DEGs were mainly divided into three expression trends. The results of the GO and the KEGG pathway enrichment analyses showed that pathways and genes related to signal transduction, osmoregulation, and metabolism may be involved in the adaptive regulation of salinity in the yellow drum. In conclusion, by identifying the candidate genes and the cellular pathways involved in salinity stress response, we provided basic transcriptome information for the further study of the salinity adaptability of the yellow drum. The genes and pathways might be valuable for further targeted studies on salinity tolerance in the yellow drum, facilitating selective breeding of salinity-tolerant yellow drum lines for the aquaculture industry.

## Data Availability

The raw data files were passed to NCBI’s Sequence Read Archive (SRA) (GenBank accession no. PRJNA736181).

## Supplementary Materials

The following are available online at https://www.mdpi.com/article/10.3390/ani11082201/s1, Table S1. Genes and gene-specific primers used for qRT-PCR. Table S2. The quality indicators of reads.
