# Supplementary material for: Transcriptome Profiling Reveals a Divergent Adaptive Response to Hyper- and Hypo-Salinity in the Yellow Drum, Nibea albiflora
Source: Animals (Basel). 2021 Jul 25;11(8):2201. doi: 10.3390/ani11082201 (PMC8388402; doi:10.3390/ani11082201)
Supplement: Supplementary file 1 [file animals-11-02201-s001.zip › animals-1285005-SI.pdf]

Table S1. Genes and gene-specific primers used for qRT-PCR.

Gene ID	Gene name	Primer sequence (5′-3′)
Nib0139610.1	Tyrosine-protein kinase	F: TGCCTTCTCCTTGTCCAT
		R: GGTCTGAGTAGTGCTGAAC
Nib0169820.1	N-alpha-acetyl transferase 40	F: GGAGATGAACGACGAGAG
		R: CTGTAGTATCTGGATGAGGAA
Nib0000930.1	Fibroblast growth factor	F: GCACGCAATGGTTCCTAA
		R: TGTCCAGACTTGTTCATAGAG
Nib0012590.1	Sodium/myo-inositol cotransporter	F: CAGGCGGTGTTGATGATT
		R: GGAAGGAGAATAGGTGAAGTT
Nib0021280.1	Histone H5	F: CGTGAAGAAGAACTACAAGG
		R: TTAACCAGCCTGAAGGATC
Nib0087920.1	Abhydrolase domain-containing protein 14B	F: CCGTCGCTGATTGTCTAC
		R: GAGTATTAAGGAAGTCTGTGAG
Nib0097010.1	Pentraxin-related protein PTX3	F: GACTGGAGGAGGTGCTAA
		R: GAGACAATGGATGGCTTCA
Nib0131210.1	Metalloproteinase inhibitor 2	F: GTTGGCAATGACATCTATGG
		R: CCTGTGACAAGATACTCCTT

Table S2. The quality indicators of reads

Sample	Total Raw Reads (M)	Total Clean Reads (M)	Clean Reads Q20(%)	Clean Reads Q30(%)
HYD10_1	51.04	50.04	98.2	94.74
HYD10_2	48.87	47.93	98.09	94.47
HYD10_3	45.09	44.09	98.03	94.36
HYD15_1	46.96	46.04	98.27	94.9
HYD15_2	49.46	48.34	98.11	94.54
HYD15_3	50.03	48.97	98.28	94.92
HYD20_1	48.5	47.44	98.37	95.17
HYD20_2	51.93	50.19	98.09	94.52
HYD20_3	53.59	52.47	98.24	94.86
HYD25_1	49.34	47.98	98.27	94.9
HYD25_2	49.86	48.62	98.39	95.23
HYD25_3	47.13	46.26	98.28	94.9
HYD30_1	44.69	43.81	98.23	94.83
HYD30_2	48.18	47.31	98.28	94.92
HYD30_3	42.36	41.33	98.02	94.35
HYD35_1	44.09	43.25	97.93	94.11
HYD35_2	52.81	51.69	98.21	94.79
HYD35_3	48.92	48.01	98.22	94.78
